# Implication of Nonalcoholic Fatty Liver Disease, Metabolic Syndrome, and Subclinical Inflammation on Mild Renal Insufficiency

**DOI:** 10.1155/2018/1835486

**Published:** 2018-04-02

**Authors:** Ga Eun Nam, Soon Young Hwang, Hye Soo Chung, Ju Hee Choi, Hyun Jung Lee, Nam Hoon Kim, Hye Jin Yoo, Ji-A Seo, Sin Gon Kim, Nan Hee Kim, Sei Hyun Baik, Kyung Mook Choi

**Affiliations:** ^1^Division of Endocrinology and Metabolism, Department of Internal Medicine, Korea University Guro Hospital, College of Medicine, Korea University, 80 Guro-dong, Guro-gu, Seoul 152-050, Republic of Korea; ^2^Department of Family Medicine, Sahmyook Medical Center, 80 Mangu-ro, Dongdaemun-gu, Seoul 02500, Republic of Korea; ^3^Department of Biostatistics, College of Medicine, Korea University, 73 Inchon-ro, Seongbuk-gu, Seoul 02841, Republic of Korea

## Abstract

**Background:**

Limited information exists about the impact of nonalcoholic fatty liver disease (NAFLD) on mild renal insufficiency. We compared the relative influence of NAFLD, metabolic syndrome (MetS), and subclinical inflammation, alone or in combination, on mild renal insufficiency.

**Methods:**

This study included 1174 Korean adults. NAFLD was diagnosed using ultrasonography. Mild renal insufficiency was defined as an estimated glomerular filtration rate (eGFR) ≥ 60 and <90 mL/min/1.73 m^2^.

**Results:**

In partial correlation analysis, several components of MetS and liver aminotransferase levels, but not high-sensitivity C-reactive protein (hsCRP), were associated with eGFR. Multivariate logistic regression analysis demonstrated the independent association of NAFLD (*P* = 0.034) and MetS (*P* = 0.018) with mild renal insufficiency, but not elevated hsCRP (*P* = 0.885). Furthermore, NAFLD without the MetS group (odds ratio (95% confidence interval) = 1.56 (1.05–2.34)) or MetS without the NAFLD group (1.82 (1.11–3.00)) was associated with mild renal insufficiency after adjusting for confounding variables. However, individuals with high hsCRP showed no relationship with mild renal insufficiency, irrespective of the existence of NAFLD.

**Conclusions:**

This study demonstrated that NAFLD and MetS are independently associated with mild renal insufficiency, whereas subclinical inflammation did not affect the risk for mild renal insufficiency in Korean adults.

## 1. Introduction

The prevalence of chronic kidney disease (CKD) has increased worldwide in accordance with the global increase in associated traditional risk factors including aging, diabetes, and hypertension [1, 2]. Thus, CKD has become a major public health problem posing substantial burden on healthcare costs [3]. Furthermore, CKD increases the risks for cardiovascular diseases (CVDs) and all-cause mortality, besides end-stage renal disease [4, 5]. Recent evidence has suggested that mild renal insufficiency is also associated with all-cause and cardiovascular mortality [4, 6]. Because of substantial morbidity and mortality, detection and management of CKD in the earlier stages and extensive evaluation of modifiable risk factors might be warranted [7].

Nonalcoholic fatty liver disease (NAFLD), as the most common chronic liver disease worldwide, has a potential to progress to cirrhosis, end-stage liver disease, or hepatocellular carcinoma [8]. NAFLD has been recognized to be linked to cardiometabolic risk factors and its prevalence is rapidly increasing concomitantly, with the global epidemics of obesity and type 2 diabetes mellitus (T2DM). NAFLD has also been reported to affect the development of extrahepatic diseases, including CVD and CKD [9, 10]. Recent studies have reported that NAFLD is associated with increased prevalence and incidence of CKD [11]. NAFLD aggravates insulin resistance, predisposing to atherogenic dyslipidemia and releasing various proinflammatory molecules that may lead to vascular and renal damage [12]. However, few data are available regarding the implication of NAFLD on mild renal insufficiency.

Metabolic syndrome (MetS) is a cluster of cardiometabolic risk factors and is well known to increase the risk for T2DM, CVD, and mortality. Recent studies have reported that MetS may foster the development of CKD [13, 14]. This might be explained by their common pathophysiology, such as insulin resistance, chronic inflammation, and oxidative stress. However, only a few studies have evaluated the impact of insulin resistance on incident CKD, yielding conflicting results [15, 16]. Chronic inflammation is known as a phenotype of CKD and is associated with the progression of renal injury as well as adverse cardiovascular outcomes [17, 18]. High-sensitivity C-reactive protein (hsCRP) is a representative biomarker of systemic subclinical inflammation [19]. However, the role of inflammation in mild renal impairment has not been adequately evaluated [18, 20–23].

The present study compared the relative influence of NAFLD, MetS, and subclinical inflammation on mild renal insufficiency. We also aimed to explore whether NAFLD accompanied by MetS or elevated hsCRP has a synergistic impact on mild renal insufficiency, in relatively healthy men and women.

## 2. Materials and Methods

### 2.1. Study Design and Participants

This study included healthy Korean men and women, aged 20–80 years old, who were self-referred and underwent routine health examinations at the health promotion center of Korea University Guro Hospital, located in Seoul, South Korea, from April 1, 2012 to August 31, 2013. Subjects who had a daily alcohol consumption ≥ 30 g for men and ≥20 g for women [24], those who had been diagnosed with viral hepatitis or other hepatic diseases, and CVD such as coronary heart disease or stroke, those who had histories of inflammatory diseases, those with drug histories of steroid or nonsteroidal anti-inflammatory drugs within 6 months, and those diagnosed with malignancies were excluded. Additionally, we excluded participants who had moderate to severe renal dysfunction (estimated glomerular filtration rate (eGFR) < 60 mL/min/1.73 m^2^). Finally, 1174 subjects were included for the data analyses. All study participants gave written informed consent, and the study protocol was approved by the Institutional Review Board at the Korea University Guro Hospital in accordance with the Declaration of Helsinki of the World Medical Association.

### 2.2. Data Collections

All participants completed detailed questionnaires including medical histories and lifestyle characteristics. Smoking status was categorized as “nonsmokers,” “ex-smokers,” and “current smokers.” Alcohol consumption was classified into two groups based on the average alcohol amount consumed per day for a month prior to the day of reporting the questionnaire: “nondrinkers” and “alcohol users” (≥1 and <30 g/day for men and ≥1 and <20 g/day for women) [24, 25]. Physical activity was categorized as “none” or “regular.” Individuals were regarded as regular physical exercisers if they exercised ≥ 3 times per week for ≥30 min per session. Trained staff performed anthropometric measurements. Body weight and height were measured with participants in light indoor clothing, and body mass index (BMI) calculated as weight (in kg) divided by height (in m) squared. Waist circumference (WC) was measured at the narrowest point between the lower border of rib cage and iliac crest at the end of exhalation. Blood pressure (BP) was measured at the upper arm by using an automatic BP-monitoring device after participants rested for at least 5 min. Blood samples were obtained after an overnight fast and immediately stored at −80°C for subsequent assays. Serum concentrations of total cholesterol, triglyceride, high-density lipoprotein cholesterol (HDL-C), low-density lipoprotein cholesterol (LDL-C), aspartate aminotransferase (AST), and alanine aminotransferase (ALT) were measured using an automatic biochemical analyzer (TBA-2000FR; Toshiba Medical Systems, Tokyo, Japan). Plasma glucose levels were measured using the glucose oxidase method, and hemoglobin A1c (HbA1c) by the high-performance liquid chromatography method. The level of hsCRP was measured using a latex-enhanced turbidimetric immunoassay (HiSens hsCRP LTIA; HBI, Anyang, Korea). Subjects were divided into low-hsCRP group (hsCRP < 1 mg/L) and high-hsCRP group (hsCRP ≥ 1 mg/L) [26]. The value for eGFR was calculated using the equation from the Modification of Diet in Renal Disease (MDRD) study: eGFR = 175 × serum creatinine^−1.154^ × age^−0.203^ × 0.742 (for females) [27]. Mild renal insufficiency was defined as eGFR ≥ 60 and <90 mL/min/1.73 m^2^ according to the categorization of the National Kidney Foundation [28].

### 2.3. Definitions of NAFLD and MetS

Abdominal ultrasonography (iU22; Philips Healthcare, Bothell, WA, USA) was performed to diagnose hepatic steatosis by a single experienced radiologist. Images were captured in a standard manner (i.e., the subject was in the supine position with the right arm raised above the head), and the degree of hepatic steatosis was categorized as “absent,” “mild,” “moderate,” or “severe,” based on an already established sonographic criteria. NAFLD was defined as the presence of more than a mild degree of hepatic steatosis [29].

MetS was defined based on the modified criteria of the National Cholesterol Education Program Adult Treatment Panel III, while for abdominal obesity, the Asian-specific WC cutoff was adopted [30, 31]. Subjects with at least three of the following components were indicated as having MetS: (i) WC ≥ 90 cm for men and ≥80 cm for women; (ii) serum triglyceride ≥ 150 mg/dL; (iii) serum HDL-C < 40 mg/dL for men and <50 mg/dL for women; (iv) systolic BP ≥ 130 mmHg, diastolic BP ≥ 85 mmHg, or treatment with antihypertensive medication; and (v) fasting plasma glucose ≥ 100 mg/dL or use of hypoglycemic agents.

### 2.4. Statistical analyses

Data were presented as the median and interquartile ranges (25–75%), or as *n* (%). The independent *t*-test for continuous variables and Chi-square test for categorical variables were performed to assess the differences in characteristics based on the presence or absence of NAFLD. The Spearman partial correlation analysis was used to assess the correlation between cardiometabolic risk factors and eGFR after adjusting for age and sex (for total subjects) or age only (for men and women). Hierarchical multivariable logistic regression analysis was performed to estimate the odds ratios (ORs) and 95% confidence intervals (95% CIs) of mild renal insufficiency according to the presence of NAFLD, MetS, and high hsCRP. Model 1 was unadjusted, while model 2 was adjusted for age, sex, smoking status, alcohol consumption, and physical activity. Variables adjusted in model 2, in addition to BMI, AST, ALT, and LDL-C, were adjusted in model 3. Data were analyzed by a professional statistician (S.Y. Hwang, PhD), one of the authors, using the Statistical Analysis System (SAS) 9.2 software for Windows (SAS Institute Inc., Cary, NC, USA). All statistical tests were two tailed, and a *P* value of <0.05 was considered statistically significant.

## 3. Results

### 3.1. Characteristics of Study Subjects

Table 1 presents characteristics of study subjects with respect to the presence of NAFLD. Among a total of 1174 subjects, 386 (32.9%) individuals had NAFLD diagnosed by ultrasonography. Subjects with NAFLD were more likely to be older (*P* = 0.018) and males (*P* < 0.001). The mean values of cardiometabolic parameters such as BMI, WC, systolic BP, diastolic BP, total cholesterol, triglyceride, LDL-C, glucose, and HbA1c as well as liver enzymes (AST and ALT) were significantly higher in subjects with NAFLD compared to those without NAFLD, whereas HDL-C levels were higher in subjects without NAFLD (*P* < 0.001). The hsCRP levels were higher in the NAFLD group than in the normal group (*P* < 0.001). Subjects with NAFLD had a lower mean eGFR level than those without NAFLD, and the proportion of subjects with mild renal insufficiency was higher in the NAFLD group than in the normal group (*P* < 0.001). As expected, subjects with NAFLD showed a higher prevalence of MetS, DM, and hypertension than those without NAFLD (*P* < 0.001). While the proportion of current smokers was significantly higher in the NAFLD group compared to those in the normal group (*P* < 0.001), proportions of alcohol users and regular exercisers were not different between the two groups.

### 3.2. Partial Correlations between Cardiometabolic Parameters and eGFR

Table 2 shows correlations between NAFLD-related cardiometabolic parameters and eGFR after adjusting for age and sex. BMI, WC, total cholesterol, triglyceride, LDL-C, AST, ALT, and glucose were inversely correlated with eGFR among all subjects. In men, BMI, WC, total cholesterol, triglyceride, and LDL-C exhibited inverse correlations with eGFR, and HDL-C was positively associated with eGFR. Total cholesterol, triglyceride, and AST were inversely correlated with eGFR in women. However, hsCRP showed no correlation with eGFR.

### 3.3. Multivariable Logistic Regression Analysis for Mild Renal Insufficiency as a Dependent Variable with Respect to the Presence of NAFLD, MetS, and High hsCRP

Table 3 presents ORs (95% CIs) of mild renal insufficiency (eGFR ≥ 60 and <90 mL/min/1.73 m^2^) according to the presence of NAFLD, MetS, and high hsCRP (hsCRP ≥ 1 mg/L). In an adjusted model of multivariable logistic regression analysis, individuals with NAFLD (OR, 95% CI = 1.44, 1.03–2.01) or MetS (OR, 95% CI = 1.54, 1.08–2.20) were associated with an increased risk of mild renal insufficiency compared to those without NAFLD or MetS, respectively. Furthermore, the NAFLD without MetS group (OR, 95% CI = 1.56, 1.05–2.34) or MetS without NAFLD group (OR, 95% CI = 1.82, 1.11–3.00) was significantly associated with mild renal insufficiency, respectively (model 3). However, individuals with elevated hsCRP levels showed no association with mild renal insufficiency irrespective of the existence of NAFLD.

## 4. Discussion

The results of the present study demonstrated that the presence of NAFLD or MetS was significantly associated with an increased risk for mild renal insufficiency. These associations did not attenuate from adjustment for confounding variables including age, sex, smoking status, alcohol consumption, physical activity, BMI, AST, ALT, and LDL-C. However, elevated hsCRP reflecting subclinical systemic inflammation was not significantly associated with mild renal insufficiency.

Increasing evidence suggests that NAFLD may increase the risk for CKD. Several cross-sectional studies have consistently shown the relationships between NAFLD and the increased prevalence of CKD independently of traditional confounding factors, both in populations with and without diabetes [32, 33]. In addition, a few longitudinal studies have evaluated that NAFLD diagnosed by ultrasonography increased the risk of CKD development [11, 34]. Moreover, some previous studies have also reported that histologically proven NAFLD is associated with lower eGFR and the histologic severity of NAFLD is positively related with renal dysfunction [35, 36]. A recent meta-analysis of these associations summarized that NAFLD was associated with an increased OR of 2.12 (95% CI = 1.69–2.66) for prevalent CKD and a hazard ratio of 1.79 (95% CI 1.65–1.95) for developing incident CKD [1]. However, the associations observed from previous studies may be inconclusive because of several improper adjustments of confounding factors. Moreover, most of the prior studies assessed the relationship of NAFLD with moderate to severe renal dysfunction, based on the eGFR cutoff of <60 mL/min/1.73 m^2^, or overt proteinuria. To the best of our knowledge, only one previous study has supported our findings, reporting that NAFLD is associated with a higher risk of mild kidney function damage (eGFR < 90 mL/min/1.73 m^2^) [37]. This previous study and the current studies that focused on mild renal dysfunction may provide additional information about the associations between NAFLD and the early stage of renal dysfunction.

The underlying mechanisms linking NAFLD and renal dysfunction have not been fully elucidated. NAFLD and CKD seem to share important obesity-associated metabolic risk factors, and these two diseases may be driven commonly by obesity-associated mechanisms including lipotoxicity, oxidative stress, enhanced proinflammatory cytokines, and renin-angiotensin-aldosterone system (RAAS) activation [38]. Expanded and inflamed visceral adipose tissues release systemic pathogenic factors including free fatty acids and proinflammatory cytokines, resulting in insulin resistance and kidney damage [1, 38–40]. Inflammatory and procoagulant factors released from a steatotic liver may be involved in the pathogenesis of CKD, while other hepatic factors including fetuin-A and fibroblast growth factor may also be related to kidney damage in patients with NAFLD [41, 42]. Meanwhile, the proposed relationship between CKD and dyslipidemia suggests a vicious cycle between NAFLD and CKD [43].

Previous studies have documented positive relationships between insulin resistance and CKD, suggesting that MetS is an important relevant risk factor for the development and progression of CKD [13, 44]. Although the mechanisms of the close association between MetS and renal damage are not fully understood, adipose tissue expansion in patients with MetS promotes chronic inflammation and oxidative stress, thereby exacerbating insulin resistance. This process may result in renal impairment by endothelial dysfunction, activation of the RAAS, and an imbalance of adipocytokines. Insulin resistance and inflammation can also contribute to microvascular remodeling and podocyte damage, leading to renal parenchymal damage and albuminuria [45]. These possible underlying mechanisms between MetS and CKD seem to be partly similar to the aforementioned mechanisms linking NAFLD and CKD. Furthermore, in patients with CKD, hepatic steatosis was reported to be closely correlated with the risk of MetS [46]. Meanwhile, an interesting observation from our study that the NAFLD group even without MetS was independently associated with mild renal dysfunction, suggests that the impact of NAFLD on mild renal insufficiency might originate from other factors independent of the components of metabolic syndrome and traditional risk factors.

hsCRP has been suggested to participate in the pathogenesis of glomerulosclerosis by inducing adhesion molecules in endothelial cells and depositing them along the walls of the glomerular capillaries. However, the data evaluating the association between hsCRP and renal function are conflicting [18, 20–23]. The current findings from relatively healthy Korean adults did not show a significant association between hsCRP and mild renal insufficiency, irrespective of the existence of NAFLD. Although several previous studies have reported an inverse correlation between GFR and inflammation, these were found predominantly in progressed stages of CKD. Biomarkers of inflammation appear to have various predictive values in different stages of CKD and thus, the pathophysiology of inflammation may be different in CKD patients with different genetic backgrounds [47]. Actually, hsCRP levels in this study seemed to have been relatively lower compared to data from Caucasian studies. Further studies on a larger scale are also needed to verify the association observed in the current study.

Several limitations of this study should be addressed. First, the cross-sectional design did not allow an establishment of causal relationships among NAFLD, MetS, inflammation, and mild renal insufficiency with certainty. Second, the samples were mostly based on a relatively healthy Asian population, therefore findings are not nationally representative or geographically diverse. Third, although the sonogram is accepted as a diagnostic tool for NAFLD, it may not be an accurate measurement for hepatic steatosis. On the other hand, a liver biopsy, which is the gold standard for the diagnosis of NAFLD, is not feasible in epidemiologic studies due to invasiveness.

## 5. Conclusions

The current study demonstrated that NAFLD or MetS was independently associated with an increased risk for mild renal insufficiency in Korean men and women. However, elevated hsCRP did not aggravate the risk for mild renal insufficiency, irrespective of the existence of NAFLD. These results may provide a novel insight into the pathogenesis and clinical implications of risk factors associated with mild renal insufficiency.

## Figures and Tables

**Table 1:** Characteristics of study subjects.

	NAFLD (−)	NAFLD (+)	*P* value
*N*	788	386	
Age (years)	52 (44, 58)	53 (47, 58)	0.018
Sex (male, %)	297 (37.7)	227 (58.8)	<0.001
BMI (kg/m^2^)	22.6 (20.8, 24.7)	25.2 (23.7, 27.3)	<0.001
WC (cm)	75 (69, 81)	84 (79, 90)	<0.001
Systolic BP (mmHg)	116 (105, 127)	122 (113, 132)	<0.001
Diastolic BP (mmHg)	75 (68, 84)	79 (72, 87)	<0.001
Total cholesterol (mg/dL)	189 (166, 214)	195.5 (171, 223)	0.007
Triglyceride (mg/dL)	81 (55, 121)	129 (91, 192)	<0.001
HDL-C (mg/dL)	57 (47, 68)	48 (41, 55)	<0.001
LDL-C (mg/dL)	116 (93.5, 141)	125 (101, 151)	<0.001
Glucose (mg/dL)	93 (88, 100)	100 (93, 109)	<0.001
HbA1c (%)	5.3 (5.1, 5.6)	5.6 (5.3, 6)	<0.001
AST (IU/L)	24 (19, 29)	27 (22, 34)	<0.001
ALT (IU/L)	18 (14, 25)	27.5 (19, 39)	<0.001
hsCRP (mg/L)	0.4 (0.2, 1.2)	0.8 (0.4, 1.8)	<0.001
Creatinine (mg/dL)	0.7 (0.6, 0.8)	0.8 (0.6, 0.9)	<0.001
eGFR (mL/min/1.73 m^2^)	103.9 (92.2, 115.9)	97.4 (86.7, 108.3)	<0.001
eGFR < 90 mL/min/1.73 m^2^	165 (20.9)	119 (30.8)	<0.001
Metabolic syndrome (%)	107 (13.6)	168 (43.5)	<0.001
Diabetes mellitus (%)	32 (4.1)	49 (12.7)	<0.001
Hypertension (%)	116 (14.7)	105 (27.2)	<0.001
Current smokers (%)	104 (13.2)	81 (21.0)	<0.001
Alcohol users (%)	362 (47.0)	171 (45.2)	0.571
Regular exercisers (%)	298 (38.1)	130 (33.9)	0.171

Data are presented as median (interquartile range) or *n* (%). BMI, body mass index; WC, waist circumference; BP, blood pressure; HDL-C, high-density lipoprotein cholesterol; LDL-C, low-density lipoprotein cholesterol; AST, aspartate aminotransferase; ALT, alanine aminotransferase, hsCRP, high-sensitivity C-reactive protein; eGFR, estimated glomerular filtration rate.

**Table 2:** Spearman partial correlations of cardiometabolic parameters with eGFR after adjusting for age and sex (total group) or age only (men and women).

	Total		Men		Women	
	*r*	*P* value	*r*	*P* value	*r*	*P* value
BMI	−0.085	0.004	−0.179	<0.001	0.009	0.829
WC	−0.105	<0.001	−0.207	<0.001	−0.018	0.658
Systolic BP	−0.004	0.903	0.014	0.756	0.002	0.954
Diastolic BP	−0.046	0.117	−0.033	0.454	−0.032	0.413
Total cholesterol	−0.120	<0.001	−0.139	0.002	−0.096	0.015
Triglyceride	−0.176	<0.001	−0.255	<0.001	−0.114	0.004
HDL-C	0.042	0.151	0.101	0.022	−0.021	0.599
LDL-C	−0.081	0.006	−0.106	0.016	−0.048	0.220
AST	−0.076	0.010	−0.033	0.447	−0.101	0.011
ALT	−0.059	0.046	−0.050	0.253	−0.053	0.176
Glucose	−0.018	0.530	−0.041	0.358	0.002	0.967
hsCRP	0.003	0.917	−0.018	0.687	0.031	0.431

eGFR, estimated glomerular filtration rate; BMI, body mass index; WC, waist circumference; BP, blood pressure; HDL-C, high-density lipoprotein cholesterol; LDL-C, low-density lipoprotein cholesterol; AST, aspartate aminotransferase; ALT, alanine aminotransferase; hsCRP, high-sensitivity C-reactive protein.

**Table 3:** Odds ratios and 95% confidence intervals of mild renal insufficiency (eGFR ≥ 60 and <90 mL/min/1.73 m^2^) with respect to the presence of NAFLD, MetS, and high hsCRP.

	Model 1^a^	Model 2^b^	Model 3^c^
NAFLD (−) (ref.)	1	1	1
NAFLD (+)	1.68 (1.28–2.22)	1.58 (1.17–2.14)	1.44 (1.03–2.01)
*P* value	<0.001	0.003	0.034
MetS (−) (ref.)	1	1	1
MetS (+)	2.22 (1.65–2.98)	1.79 (1.30–2.46)	1.54 (1.08–2.20)
*P* value	<0.001	<0.001	0.018
hsCRP < 1 mg/L (ref.)	1	1	1
hsCRP ≥ 1 mg/L	1.14 (0.86–1.51)	1.09 (0.80–1.49)	0.98 (0.71–1.34)
*P* value	0.375	0.571	0.885
NAFLD (−), MetS (−) (ref.)	1	1	1
NAFLD (+), MetS (−)	1.76 (1.23–2.51)	1.60 (1.09–2.34)	1.56 (1.05–2.34)
NAFLD (−), MetS (+)	2.93 (1.90–4.52)	2.03 (1.26–3.25)	1.82 (1.11–3.00)
NAFLD (+), MetS (+)	2.39 (1.64–3.47)	2.08 (1.39–3.11)	1.88 (1.17–3.01)
*P* value	<0.001	<0.001	0.017
NAFLD (−), hsCRP < 1 mg/L (ref.)	1	1	1
NAFLD (+), hsCRP < 1 mg/L	1.74 (1.23–2.47)	1.54 (1.05–2.25)	1.42 (0.95–2.12)
NAFLD (−), hsCRP ≥ 1 mg/L	1.10 (0.75–1.61)	0.99 (0.65–1.50)	0.93 (0.61–1.42)
NAFLD (+), hsCRP ≥ 1 mg/L	1.76 (1.18–2.60)	1.68 (1.10–2.57)	1.43 (0.89–2.30)
*P* value	0.003	0.024	0.182

eGFR, estimated glomerular filtration rate; NAFLD, nonalcoholic fatty liver disease; MetS, metabolic syndrome; hsCRP, high-sensitivity C-reactive protein. ^a^Model 1 was unadjusted. ^b^Model 2 was adjusted for age, sex, smoking status, alcohol consumption, and physical activity. ^c^Model 3 was adjusted for age, sex, smoking status, alcohol consumption, physical activity, body mass index, aspartate aminotransferase, alanine aminotransferase, and low-density lipoprotein cholesterol.

## Data Availability

The datasets analyzed during the current study are not publicly available due to the sensitive nature of the data and the consent agreements signed by the participants, but are available from the corresponding author (Dr. Kyung Mook Choi) on reasonable request.
